# Impaired sustained attention in groups at high risk for antisocial personality disorder: A contingent negative variation and standardized low-resolution tomographic analysis study

**DOI:** 10.3389/fnhum.2022.925322

**Published:** 2022-11-23

**Authors:** Muzhen Guan, Lifang Ma, Yifang Zhu, Yang Liao, Lingwei Zeng, Shengjun Wu, Ke Men, Xufeng Liu

**Affiliations:** ^1^Department of Psychiatry, Xi’an Medical University, Xi’an, Shaanxi, China; ^2^School of Military Medical Psychology, Air Force Medical University, Xi’an, Shaanxi, China; ^3^State Key Laboratory of Military Stomatology & National Clinical Research Center for Oral Diseases & Shaanxi Key Laboratory of Stomatology, Department of Operative Dentistry and Endodontics, School of Stomatology, Air Force Medical University, Xi’an, Shaanxi, China; ^4^Department of Thoracic Surgery, Tangdu Hospital, Air Force Medical University, Xi’an, Shaanxi, China; ^5^Air Force Medical Center, Air Force Medical University, Xi’an, China

**Keywords:** conduct disorder, antisocial personality, contingent negative variation, event-related potential, attention

## Abstract

**Objective:**

This study aimed to explore the characteristics of contingent negative variation (CNV) in groups at high risk for antisocial personality disorder.

**Materials and methods:**

A classic CNV paradigm was used to compare the characteristics of attention maintenance among a group of individuals with conduct disorder (CD group; *n* = 27), a group of individuals with antisocial personality traits (AP; *n* = 29), a group of individuals with conduct disorder and antisocial personality traits (CD + AP group; *n* = 25), and a group of healthy controls (CG group; *n* = 30), to examine the characteristics of the amplitude and latency of CNV in different processing stages.

**Results:**

Results of the event-related potential analysis were as follows: The mean amplitude analysis between 500 and 1,000 ms revealed that the mean CNV amplitudes in the CD + AP group (-1.388 ± 0.449 μV, *P* < 0.001) were significantly lower than that in the CG group (-4.937 ± 0.409 μV). The mean amplitude analysis between 1,000 and 1,500 ms revealed that the mean CNV amplitude in the CD + AP group (-0.931 ± 0.646 μV) was significantly lower than that in the CG group (4.809 ± 0.589 μV, *P* < 0.001). The mean amplitude analysis between 1,500 and 2,000 ms revealed that the mean CNV amplitude in the CG group (3.121 ± 0.725 μV) was significantly higher than that in the CD + AP group (-0.277 ± 0.795 μV, *P* = 0.012), whereas the mean CNV amplitude in the CD + AP group was not significantly different in the AP group (*P* = 0.168) and CD group (*P* > 0.05). Source localization results indicated altered activity in frontal-temporal regions.

**Conclusion:**

The CNV amplitude characteristics in the CD + AP group and AP group were more consistent and fluctuated around the baseline, indicating the absence of attention maintenance resulted in impairments in attention allocation and motor preparation in the CD + AP group and AP group.

## Introduction

Impulsiveness is a primary characteristic of conduct disorder (CD) and antisocial personality disorder (APD) (Fairchild et al., 2019; Marzilli et al., 2021). Delk et al. (2020) integrated biological, psychological, and sociological perspectives and observed that people with impulsive traits tended to respond rapidly and in an unplanned manner to internal and external stimuli, irrespective of whether these responses had a negative impact on themselves or others (Mackey et al., 2017). The primary characteristics of impulsiveness are swiftness, lack of planning, and inappropriate behavior in a specific environment (Jupp et al., 2020). Impulsiveness involves a complex neural mechanism, a common feature of many mental illnesses, such as APD and CD (Taylor and Iacono, 2007; Garofalo et al., 2018).

The mechanism of behavioral impulses involves a complex neural mechanism and is related to many cognitive processes (Beauchaine et al., 2017). Attentional impulsiveness reflects the degree of individual attention and cognitive stability, namely, the quality of attention maintenance (Patrick, 2021). However, attention maintenance in APD has been scarcely studied. Tools and tasks, such as the Barratt Impulsiveness Scale, the Iowa Gambling Task, and the Immediate Memory Task have been used to compare differences in impulsiveness between groups with APD and healthy controls and have revealed that groups with APD were more likely to engage in unplanned and impulsive behavior and to have weakened attention maintenance (Raine, 2018; Holzer et al., 2022). Deficiencies in attention maintenance in groups with APD may be the main mechanism leading to behavioral impulses (Gil-Fenoy et al., 2018).

The impulsive behavior of APD is closely related to crime (Piotrowska et al., 2015; Tielbeek et al., 2017), and therefore high-risk antisocial groups have aroused widespread concern among researchers (Fellmeth et al., 2018; Frances et al., 2018). Researchers agree that groups with CD are at high risk for APD (Fazel et al., 2012). However, longitudinal studies have found that only approximately 40% of adolescents with CD developed APD in adulthood (Powers et al., 2016), while the remaining adolescents automatically broke away from antisocial behavior in adulthood (Wesseldijk et al., 2018). Moffitt et al. reported that the development of antisocial personality could be explained in terms of the “stability theory”, that it is temporal (Moffitt et al., 2002). Hodgins proposed that antisocial personalities and those at high risk are homogeneous (Hodgins and Côté, 1993). Based on previous studies, the present study further proposed the characteristics of groups at high risk for APD and that the development of antisocial personality had both “temporal” and “directional” characteristics; that is, groups with CD and antisocial personality traits could be at high risk for APD.

Attention maintenance is closely related to focusing on a particular task for a certain amount of time (Li et al., 2017). Event-related potential (ERP) technology has high time resolution capabilities (Guan et al., 2016). In ERP, contingent negative variation (CNV) gives a warning stimulus (S1 [prompt signal]) before a command stimulus (S2 [command signal]) during an operation task and records a long-lasting negatively deflected slow potential in the parietal area, which is connected with complex psychological factors such as preparation, expectation, attention, motivation, and awakening, thus being a suitable method for studying attention maintenance (John et al., 2019). Contingent negative variation consists of two components, an early component, and a late component. The early processing of CNV, induced by prompt signals, is related to perceptual processing and reflects the evaluation of predictive information. However, the late processing of CNV, induced by command signals, is related to behavioral and cognitive preparation. Contingent negative variation is positively correlated with attention (Vallet et al., 2019) and is closely connected with attention and concentration in the cerebral cortex. The CNV amplitude was shown to decrease when the subject was distracted (Yancey et al., 2021). Functional magnetic resonance imaging (fMRI) studies have demonstrated that CNV in the frontal lobe is a sensitive indicator for attention and distraction (Blair et al., 2018).

It has been found that adolescents with CD had significantly lower attention maintenance than their non-CD peers (Batky et al., 2020). However, few studies have examined attention quality differences in patients with CD and APD. In this present study, the subjects were juvenile delinquents with CD and high risk for APD (Guan et al., 2015) and adult criminals with AP. The first-class classic CNV paradigm was used to explore the attention maintenance ability of groups at high risk for APD and the ERP characteristics of early and late processing in the time course of attention maintenance. The CNV characteristics of juvenile delinquents with AP were compared with those of juvenile delinquents with CD to electrophysiologically explore attention maintenance and its mechanism in groups at high risk for APD.

## Materials and methods

### Subjects

A total of 1,103 male juvenile delinquents were selected from a juvenile detention facility. First, the Personality Diagnostic Questionnaire-4th edition plus (PDQ-4+) was used for preliminary screening. The PDQ-4 consists of two components: a self-reported questionnaire and an interview-based Clinical Significance Scale (CSS), and the evaluation of AP consists of two ways: PDQ-4+ and a SCID-II interview. Then, those with a score > 3 points were interviewed according to the structural outline of the Diagnostic and Statistical Manual of Mental Disorders, Fifth Edition (DSM-V). The 52 juvenile delinquents screened were divided into two groups. One group of subjects consisted of juvenile delinquents with CD (CD group). In that group, there were 26 subjects aged 15–17 years (mean ± standard deviation [SD], 15.70 ± 0.95), with a prison term of 2–5 years (mean ± SD, 3.96 ± 1.02), a mean (± SD) PDQ-4 + score of 3.63 ± 0.79, and junior high school education. They all met the DSM-V criteria for CD.

The second group consisted of juvenile delinquents with CD and antisocial personality traits (CD + AP group), classified as the high-risk group with APD in this present study. This group consisted of 25 subjects aged 15–17 years (mean ± SD, 15.52 ± 0.87), with a prison term of 2–5 years (mean ± SD, 3.88 ± 0.83), a mean (± SD) PDQ-4 + score of 6.6.0 ± 0.91, and junior high school education. They all met the DSM-IV criteria for CD and had a minimum of three antisocial behaviors in APD.

A total of 648 violent criminals were selected from prison. The PDQ-4 + was used for preliminary screening, and those with a score > 3 points were interviewed following the structural outline of DSM-V, and violent criminals were considered as the antisocial personality group (AP group). The group consisted of 29 subjects who met the diagnostic criteria for CD in DSM-V, aged 18–26 years (mean ± SD, 21.89 ± 2.48), had a prison term of 3–15 years (mean ± SD, 8.93 ± 3.03), a mean (± SD) PDQ-4 + score of 6.10 ± 1.48, and educational background of junior high school.

A healthy control group (CG) was also set up. Thirty subjects were selected from an enrolled youth cohort, were aged 16–20 years (17.70 ± 1.24), had an educational background of junior high school, scored ≤ 3 points on the PDQ-4 + (mean ± SD, 1.51 ± 0.68), and without a history of criminal conduct.

None of the four groups of subjects had a history of alcohol or drug abuse, brain trauma, or suicide. Neither the individuals nor their relatives had been diagnosed with mental illness or had a history of taking psychiatric drugs. All the subjects read and understood the experimental procedures and precautions, and signed the informed consent form before the start of the study. This study was reviewed and approved by the Ethics Committee of the First Affiliated Hospital of the Air Force Medical University (Approval No.: KY20182047-F-1) and was registered in the Chinese Clinical Trial Registry (Registration No.: ChiCTR1800019761).

### Experimental materials

The PDQ-4 + is a self-reported questionnaire used to screen for personality disorders (Zhou et al., 2019). Huang et al. (1998). conducted trial testing in healthy Chinese population samples and found that the PDQ-4 + (Chinese version) had good validity and reliability and could be used as a desirable screening tool for personality disorders Huang et al. (1998). The antisocial personality subscale includes 8 items, among which the eighth item include 15 sub-items, of which 3 are positive sub-items, with an entry score of 1 point.

### Experimental procedures

**FIGURE 1:**
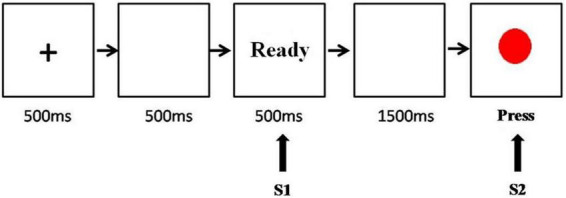
The temporal sequence of events in the contingent negative variation task.

A first-class classic CNV experimental paradigm was used (Figure 1). All experimental stimuli were presented in the center of the computer screen. The CNV-induced stimulus mode consisted of two successive visual stimuli: S1 and S2. The first stimulus, S1, was a prompt signal with the prompt word “Ready”, whereas the second stimulus, S2, was a red dot, the command signal. After receiving S1, the subjects began to expect the appearance of S2. Once the red dot appeared, they were required to immediately press the “/” key on the keyboard. If the red dot did not appear, they were required to proceed to the next experiment. The interval between S1 and S2 was 2 s and between S1 and the next S2 was 3–6 s. The experiments included one exercise and four formal experiments. In the exercise, there were 10 dots and 10 blank screens, whereas, in each formal experiment, there were 15 dots and 15 blank screens. All subjects completed the exercise first. After the experimenter confirmed that the subjects understood and could complete the experimental tasks correctly, the formal experiments were started and continuous brain waves were recorded using an electroencephalogram (EEG). During the experiments, the subjects were requested to relax, stay still, blink, and move their eyes as little as possible.

### Electroencephalogram recording

The experimental device was a 32-lead ERP workstation produced by Compumedics Neuroscan (Charlotte, NC, USA). A 32-lead Ag/AgCl electrode cap was used to record continuous brain waves (Compumedics Neuroscan). The reference electrode was placed on the tip of the nose, and the grounding electrode was placed on the forehead (grand, FPz). Four electrodes of the horizontal and vertical electrooculograms were chosen to record eye movements, with the horizontal electrooculogram placed 10 mm outside both eye corners and the vertical electrooculogram above and below the left orbit. The electrode impedance was less than 5 kΩ, had a sampling rate of 500 Hz, and a bandpass of 0.05–100 Hz. The electroencephalogram segment was 3,500 ms in total, from 500 ms before stimulation to 3,000 ms after stimulation, and 500 ms before stimulation was chosen for baseline correction. When the amplitude was above ± 100 μV, it was viewed as an artifact and automatically eliminated in the superposition.

### Standardized low-resolution tomography analysis

Based on the scalp-recorded electrical potential distribution, standardized low-resolution tomographic analysis (sLORETA)^1^ was used to compute the cortical 3D distribution of current density for a different time window of CNV between the CD + AP group and control group. In sLORETA, the intracerebral volume was partitioned in 6,239 voxels at a 5 mm spatial resolution, and the standardized current density at each voxel was then calculated in a realistic head model. To identify possible differences in the brain, the built-in voxel-wise randomization tests (5,000 permutations) based on statistical non-parametric mapping (SnPM) corrected for multiple comparisons were performed. Voxels with significant differences (corrected *p*-value < 0.05) were located in the MNI brain and Brodmann areas provided by the software.

### Statistical analysis

Behavioral data analysis was performed to determine the response time and accuracy of key pressing when the red dot appeared. Analysis of variance (ANOVA) was used to compare the differences among the CG, CD, CD + AP, and AP groups. In the ERP data analysis, the amplitudes and latencies of C3, Cz, and C4 electrodes were analyzed because the amplitudes of CNV components were the largest in the central parietal area. The analysis periods of CNV were 500–1,000 ms, 1,000–1,500 ms, and 1,500–2,000 ms, which were selected based on the theory of Rohrbaugh (Rohrbaugh et al., 1976) and Bauer’s method (Bauer, 2001). The average amplitude measurement method was adopted to perform two-way repeated measures ANOVA for statistical comparisons among the above three periods, in which electrodes (C3, Cz, and C4) were variables within the groups, whereas groups (CG group, CD group, CD + AP group, and AP group) were variables between groups.

## Results

### Behavioral analysis

ANOVA was used to analyze the differences in response time and accuracy in the classic CNV paradigm among the CG, CD + AP, CD, and AP groups. The results showed a significant difference in accuracy among the four groups (*F* [3, 107] = 4.628, *P* = 0.004). The accuracy of the CG group (94.31%) was significantly higher than the CD + AP group (92.10%, *P* = 0.012), CD group (92.19%, *P* = 0.014), and AP group (91.38%, *P* = 0.001), but no significant difference was observed among the CD + AP group, CD group, and AP group (*P* > 0.05). Response time analysis showed a significant difference among the four groups (*F* [3, 107] = 40.425, *P* < 0.001). The response time of the CG group (379.33 ± 20.35 ms) was significantly lower than the CD + AP group (461.36 ± 43.95 ms, *P* < 0.001), CD group (465.78 ± 35.59 ms, *P* < 0.001), and AP group (461.00 ± 39.32 ms, *P* < 0.001), while no significant difference was observed among the CD + AP group, CD group, and AP group (*P* > 0.05).

### Event-related potential analysis

**FIGURE 2:**
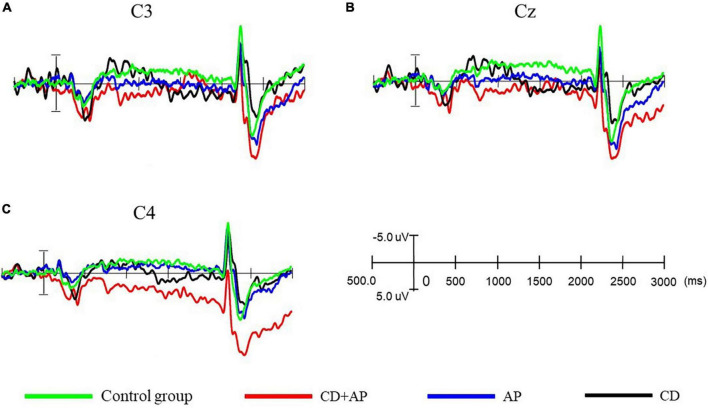
Comparison of contingent negative variation amplitudes and latencies at different electrodes among the three groups. **(A)** Comparison of contingent negative variation amplitude and latency at the C3 electrode, **(B)** at the Cz electrode, and **(C)** at the C4 electrode among the three groups.

**FIGURE 3:**
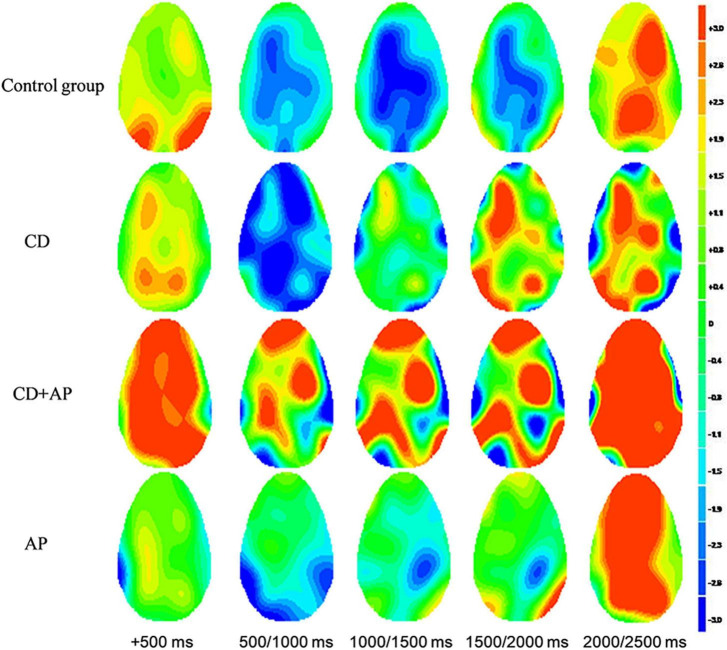
The topographic map of contingent negative variation components among the four groups.

In the CNV experiment, both the CD group and CG group showed appreciable CNV, whereas the CD + AP group and AP group did not. The temporal and spatial distribution of CNV components is shown in the waveforms of C3, Cz, and C4 (Figures 2 and 3).

Repeated measure ANOVA was used to statistically analyze the mean CNV amplitude between 500 and 1,000 ms. The results showed that the main effect of the group was significant (*F* [3, 107] = 12.565, *P* < 0.001, partial η^2^ = 0.261). Further analysis showed that the mean CNV amplitudes in the CD + AP group (-1.388 ± 0.449 μV) were significantly lower than that in the CG group (-4.937 ± 0.409 μV, *P* < 0.001) and the CD group (-3.943 ± 0.432 μV, *P* = 0.003), while there was no significant difference in the mean CNV amplitude between the CD + AP group and the AP group (*P* = 0.130; Table 1). Interactive effects were observed in the electrode × group (*F* [6, 214] = 3.812, *P* = 0.001, partial η^2^ = 0.097). Further analysis showed that at the Cz electrode, the mean CNV amplitudes in the CD + AP group (-1.410 ± 0.570 μV, *P* < 0.001), were appreciably lower than that in the CG group (-4.837 ± 0.520 μV; Table 1).

**TABLE 1:** Comparison of mean contingent negative variation amplitudes among the three groups (M ± SD).

Groups	Average amplitude of 500–1,000 ms (μV)	Average amplitude of 1,000–1,500 ms (μV)	Average amplitude of 1,500–2,000 ms (μV)
**Control group**			
C3	–5.241 ± 0.436	–4.432 ± 0.420	–3.483 ± 0.812
Cz	–4.837 ± 0.520	–4.552 ± 0.634	–3.065 ± 0.694
C4	–4.733 ± 0.271	–5.443 ± 0.833	–2.815 ± 0.669
**CD + AP group**			
C3	–1.652 ± 0.413	–0.648 ± 0.460	–0.145 ± 0.903
Cz	–1.410 ± 0.570	–1.456 ± 0.695	–0.418 ± 0.672
C4	–1.102 ± 0.360	–0.688 ± 0.912	–0.268 ± 0.810
**CD group**			
C3	–2.094 ± 0.506	–2.970 ± 0.443	–1.529 ± 0.717
Cz	–5.118 ± 0.427	–3.400 ± 0.669	–0.831 ± 0.924
C4	–4.617 ± 0.363	–3.267 ± 0.878	–0.760 ± 0.654
**AP group**			
C3	–2.109 ± 0.393	–1.572 ± 0.427	–2.327 ± 0.615
Cz	–2.710 ± 0.529	–2.697 ± 0.645	–3.042 ± 0.829
C4	–3.623 ± 0.326	–2.743 ± 0.847	–2.707 ± 0.770

M, mean; SD, standard deviation; CD, conduct disorder; AP, antisocial personality.

Repeated measure ANOVA was used to statistically analyze the mean CNV amplitude between 1,000 and 1,500 ms. The results showed that the main effect of the group remained significant (*F* [3, 107] = 6.977, *P* < 0.001, partial η^2^ = 0.164). Further analysis showed that the mean CNV amplitude in the CD + AP group (-0.931 ± 0.646 μV) was significantly lower than that in the CG group (4.809 ± 0.589 μV, *P* < 0.001; Table 1). Moreover, the main effects of different electrodes were also significant (*F* [2, 214] = 4.162, *P* = 0.017, partial η^2^ = 0.037). Further analysis showed that at the C3, Cz, and C4 electrodes, the mean CNV amplitude in the CD + AP group was significantly lower than that in the CG and CD group but not significantly different from that in the AP group (Table 1).

Repeated measures ANOVA was used to statistically analyze the mean CNV amplitude between 1,500 and 2,000 ms. The results showed that the main effect of the group was significant (*F* [3, 107] = 11.193, *P* < 0.001, partial η^2^ = 0.239). The mean CNV amplitude in the CD + AP group (-0.277 ± 0.795 μV, *P* = 0.012) was significantly lower than that in the CG group (3.121 ± 0.725 μV, *P* < 0.001), but the mean CNV amplitude in the CD + AP group was not significantly different from that in the AP group (*P* = 0.168) and CD group (*P* > 0.05; Table 1).

### Standardized low-resolution tomographic analysis results

**FIGURE 4:**
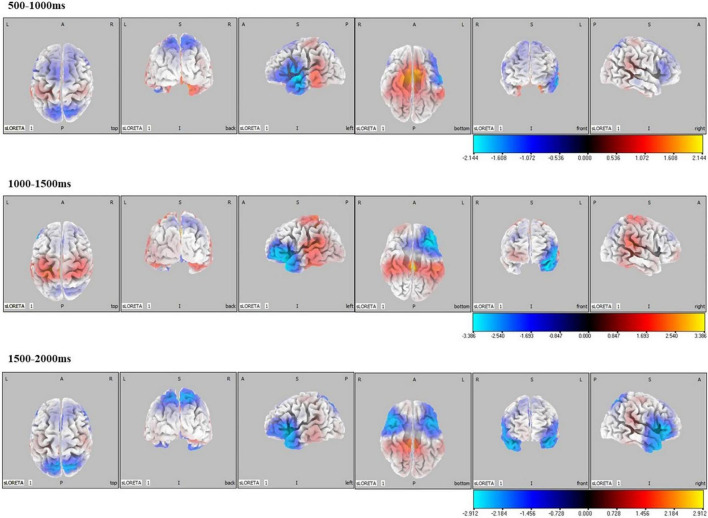
Standardized low-resolution tomographic analysis statistical non-parametric maps comparing the current density of CD + AP group and control group for significant differences in different periods of contingent negative variation.

Significant differences in brain activation were found in the different periods of CNV. Compared with the control group, in the CD + AP group, activations in the middle temporal gyrus and superior temporal gyrus were reduced in the 500–1,000 ms time window; different brain areas in the inferior frontal gyrus and superior temporal gyrus were reduced in the 1,000–1,500 ms and 1,500–2,000 ms time window, while the precuneus was increased in the 1,000–1,500 ms time window (Table 2 and Figure 4).

**TABLE 2:** Significant standardized low-resolution tomographic analysis (sLORETA) results in different time window of contingent negative variation between the CD + AP group and the control group.

Anatomical regions	MNI coordinates (x, y, z)	*t* value
**500–1,000 ms**		
Middle temporal gyrus (BA21)	–60, 0, –5	–1.934*
Superior temporal gyrus (BA22)	–60, 0, –10	–1.834*
**1,000–1,500 ms**		
Inferior frontal gyrus (BA47)	–40, 25, –10	–3.386**
Superior temporal gyrus (BA38)	–35, 20, –25	–3.304**
Postcentral (BA7)	–5, –35, 45	3.362**
**1,500–2,000 ms**		
Inferior frontal gyrus (BA47)	45, 20, 0	–2.820**
Superior temporal gyrus (BA38)	–45, 10, –10	–2.683**

*t* values correspond to *p < 0.05 and **p < 0.01, corrected for multiple comparisons of non-parametric permutation test. CNV, contingent negative variation; CD, conduct disorder; BA, Brodmann area; MNI, Montreal Neurological Institute.

## Discussion

This study investigated sustained attention in the CD + AP group with a standard CNV paradigm, and the obtained results may shed light on the association between antisocial behavior and attention processing in patients with CD + AP. Consistent with our hypothesis, the amplitudes of CNV in different periods were all reduced in the CD + AP group compared with the control group, CD group, and AP group. Source localization results demonstrated that compared with the control group, the frontal-temporal gyrus in the CD + AP group was less activated in the early, middle, and late CNV. These findings indicated that the changes in the brain activity in the frontal and temporal regions might be the essential mechanisms underlying sustained attention dysfunction in patients with CD + AP.

Research using tools and tasks, such as the Barratt Impulsiveness Scale (BIS-11), Iowa Gambling Task (IGT), and Immediate Memory Task (IMT) have provided evidence showing that groups with APD have unexpected and more impulsive behavior and weakened attention maintenance (Holzer et al., 2022). The Cambridge Neuropsychological Test Assessment Battery (CNTAB) was adopted to examine and compare the characteristics of cognitive function between patients with antisocial personalities and psychopathy. The results showed that the attention function of patients with AP was impaired (Nasaescu et al., 2020). Electroencephalogram studies on attention maintenance in APD and CD are rare (Rafiey et al., 2020). The CNV amplitude is a valuable indicator of attention. Walter et al. successively confirmed that CNV might reflect the time taken for processing (Walter, 1968), indicating that CNV amplitude may reflect the anticipatory attention and motor preparation to react to the forthcoming signal, whereby with time, more accumulation represents greater processing intensity; thus, higher amplitude.

For the behavioral assessment of this current study, the subjects were tasked to wait for the red dot to appear after seeing the visual signal “Ready.” If a blank screen appeared after preparation, the subjects did not need to respond. They were required to press the key only when seeing the red dot. Although the task was simple and the accuracy of the four groups was above 90%, the accuracy of the CG group was significantly higher than that of the other three groups. Comparisons of the response time of the four groups showed that the response times of the CD + AP group, CD group, and AP group were longer than the CG group, indicating that attention maintenance ability decreased with time.

In the ERP results of this present study, an early CNV was believed to be related to arousal and attention orientation and associated with the warning signal (Brunia, 1999). The early preparatory processes of the CD + AP group were different from the CD group and the AP group. The discrepancy in the early CNV may reflect the differences in top-down attentional mechanisms (Boland et al., 2018). In the AP group and the CD + AP group, we found no increase in amplitude from the appearance of the prompt signal to the appearance of the command signal. Therefore, the attention maintenance of the AP group and the CD + AP group was worse, which was almost unavailable. The preparation for or control over behavior might not be expected in the processing of time perception, leading to impulsive behavior (Moffitt, 2018).

The amplitudes of CNV in the AP group and the CD + AP group were reduced in the middle and late stages, suggesting the possibility of impaired motor preparation. In this present study, we found that the AP group might be homogeneous with the high-risk group in terms of antisocial personality (CD + AP group). In both the AP and CD + AP groups, we found that antisocial behavior was associated with impairment in early, middle, and late preparatory processes. More importantly, reduced late CNV appeared to be related to poorer behavioral performance in motor execution (Moore et al., 2017), which might be associated with the degraded motor preparation in the CD + AP group resulting in the deterioration of planning of movement sequence (Kersten et al., 2017).

Source localization results of the CD + AP group demonstrated that activation of the middle temporal gyrus and superior temporal gyrus was reduced in the early preparatory process, while activation of the inferior frontal gyrus and superior temporal gyrus were attenuated and postcentral was increased in the middle and late processes. Clinical and experimental data have implicated the middle temporal gyrus and superior temporal gyrus as important cortical regions in the processing of socially relevant stimuli, such as gaze following, eye direction, and head orientation (Hopkins et al., 2014), which is associated with attention (Mundy and Newell, 2007). The inferior frontal gyrus is a part of the attention network for top-down attentional control (Cazzoli et al., 2021). The postcentral gyrus is activated while preparing for initiating movements (Hanakawa et al., 2003; Ellis et al., 2021). Thus, the source localization results support the interpretation of CNV in previous studies that the early, middle, and late CNV were related to top-down attention control and motor preparation, respectively.

Although some important findings were shown in this study, there were several limitations that should be mentioned. First, the sample of this study consisted of juvenile delinquents treated at the First Affiliated Hospital of Air Force Medical University and violent adult criminals from prison. Second, the sample size was quite limited, and no power analysis was performed prior to study inclusion. The subjects were recruited based on the number of available participants; thus, the significance of the results based on the current sample size should be validated in future more extensive cohort studies. Third, the prevalence of PDs might be underestimated by using the PDQ-4 + as a screening test. Although this self-report questionnaire was demonstrated to have a high sensitivity (0.89), some participants with false negative results might have been excluded. Moreover, the sensitivity of the PDQ-4 + varies by diagnosis (Yang et al., 2000), which could also impact the diagnosis on different levels. Thus, to minimize the influence of misdiagnosis, follow-up studies would be needed.

In conclusion, the present study adopted a classic CNV paradigm to investigate the characteristics of attention maintenance in antisocial behavior. According to the results, subjects from the AP group and CD + AP group demonstrated fluctuated early and late CNV amplitudes around the baseline, indicating impairments in attention allocation, attention maintenance, and motor preparation. These also implied an association between antisocial behavior and deficits in cognition, such as preparatory processes. Additionally, we also observed that the early visual processing of CNV was in the temporal region, whereas the middle and late CNV amplitudes were related to the expectation and preparation of action, with the frontal-temporal lobe mainly activated. Furthermore, the present study showed that CNV was a sensitive indicator to distinguish high risk for AP and CD, thus laying a foundation for further examination of high-risk groups with antisocial personality traits.

## Data availability statement

The raw data supporting the conclusions of this article will be made available by the authors, without undue reservation.

## Ethics statement

This study has been reviewed and approved by the Ethics Committee of the First Affiliated Hospital of Air Force Medical University (Approval Document No.: KY20182047-F-1) and has been registered in the Chinese Clinical Trial Registry (Registration No.: ChiCTR1800019761). Written informed consent to participate in this study was provided by the participants’ legal guardian/next of kin.

## Author contributions

MG, LM, and YZ contributed equally to this study. XL, MG, YZ, and LM conceived and designed the experiments. YL, YZ, LZ, and SW performed the experiments. SW, LM, and KM analyzed the data. All authors conceived the project and modified the manuscript, read and approved the final manuscript.

## Footnotes

1
http://www.uzh.ch/keyinst/loreta.htm
